# Smart wearables for school-based physical activity promotion: K–12 physical education teachers’ continuance intention and adoption pathways

**DOI:** 10.3389/fpubh.2026.1756723

**Published:** 2026-06-18

**Authors:** Jin Zhao, Xianguo Liu, Jinhai Liu

**Affiliations:** 1 The Faculty of Education, Henan Normal University, Xinxiang, China; 2 Dongdajie Primary School, Xinmi, China; 3 Henan Provincial Collaborative Innovation Center for Intelligent Education, Henan Normal University, Xinxiang, Henan, China; 4 College of Physical Education, Henan Normal University, Xinxiang, China; 5 School of Mathematics and Statistics, Henan Normal University, Xinxiang, China

**Keywords:** perceived data privacy, physical education teachers, self-efficacy, smart wearable devices, task-technology fit model, unified theory of acceptance and use of technology model

## Abstract

**Introduction:**

Smart wearable devices are increasingly used in physical education to support data-driven monitoring and feedback. However, limited research has examined why K–12 physical education teachers continue using these devices. This study investigates teachers’ continuance intention by integrating the Task–Technology Fit model and the Unified Theory of Acceptance and Use of Technology, with self-efficacy and perceived data privacy added as extended variables.

**Methods:**

Using convenience sampling, 755 valid questionnaires were collected from K–12 physical education teachers, including 500 male teachers (66.22%) and 255 female teachers (33.78%). The model was tested using covariance-based structural equation modeling (CB-SEM), and fuzzy-set qualitative comparative analysis (fsQCA) was used to identify configurations leading to high and low continuance intention.

**Results:**

The CB-SEM results showed that task characteristics significantly predicted task–technology fit (*β* = 0.916, *p* < 0.001), whereas technological characteristics did not. Task–technology fit was the strongest predictor of continuance intention (*β* = 0.504, *p* < 0.001), followed by performance expectancy, social influence, self-efficacy, and effort expectancy. Perceived data privacy had a significant negative effect. The fsQCA identified four configurations leading to high continuance intention and five leading to low continuance intention, indicating that continued use results from multiple combinations of task-related, technological, psychological, and social conditions.

**Discussion:**

This study advances understanding of teachers’ technology adoption by integrating symmetric and asymmetric analytical approaches within a unified theoretical framework. The findings highlight the central role of task–technology fit and show that continuance intention is not determined by a single factor, but by multiple interacting conditions. These results provide practical implications for policymakers, school administrators, and technology developers seeking to support the sustainable integration of smart wearable devices into physical education and school-based physical activity promotion.

## 1 Introduction

Technological innovation has become a major driver of educational transformation and sustainable development. UNESCO’s Global Education Monitoring Report 2023 identifies technology as a key enabler of Sustainable Development Goal 4 and calls for inclusive digital education policies (1). In this context, smart wearable devices—representing the forefront of emerging technologies—have enabled deeper human–computer interaction, driven by advances in artificial intelligence. These devices provide novel approaches for educational transformation (2). Supported by Internet of Things (IoT) technologies (3), virtual reality (VR) applications (4), and the emerging metaverse ecosystem (5), smart wearables enable a “monitor–analyze–feedback–optimize” instructional model. This model significantly broadens the scope of smart wearables in educational contexts. These technologies not only enhance the accuracy and interactivity of teaching but also have significant potential to reshape the structure and pedagogical approaches of traditional physical education classrooms. With advantages such as portability, multifunctionality, and increasing accessibility, smart wearable devices hold considerable promise for future integration into educational settings (6).

Numerous studies have shown that smart wearable devices provide instant feedback, dynamic assessment, and personalized instruction, thereby enriching instructional resources (7) and enhancing interaction in physical education (8). Currently, smart wearables are applied in physical education mainly in three areas. First, as health intervention tools, they have proven effective in promoting moderate-to-vigorous physical activity (MVPA) across various populations, including preschool children (7), adolescents (9), college students (10), older adults, and cancer patients (11). Second, AI recognition systems built on multi-source data are used to detect behaviors and track learning processes, supporting self-training and fine-tuning mechanisms. In addition, self-supervised AI recognition systems based on multi-source data have been developed to detect behaviors and track learning processes. These systems employ pre-training and fine-tuning techniques and have been applied in several large-scale research projects (12, 13); Third, smart wearables support the acquisition and improvement of motor skills. By integrating intelligent systems for motion recognition and feedback optimization, these technologies can significantly improve learners’ athletic performance. For example, the WISER model, which provides multiple forms of feedback, has been applied to improve badminton skills (14). Similarly, smart bracelets have been shown to improve students’ shooting accuracy (3).

Despite their functional potential, concerns about high costs, data privacy, and the lack of systematic training hinder the sustained use of smart wearables in education (3, 15). In the integration of educational technology, teachers—central agents in curriculum implementation—play a pivotal role, and their willingness to adopt technology directly influences the adoption of smart wearables (16). Understanding physical education teachers’ willingness to continue using smart wearables is essential for education authorities seeking to develop supportive policies that promote digitally driven physical education. Studies indicate a clear divide in physical education teachers’ attitudes toward wearable technology. Some acknowledge its benefits in improving efficiency and teaching quality (4, 17), whereas others argue that it may weaken the humanistic and emotional dimensions of classroom interaction (18, 19).

These issues suggest that the effective use of smart wearables depends not only on technical performance but also on teachers’ acceptance and continuance intention. Recent educational technology research further indicates that teachers’ digital technology integration cannot be fully explained by technology availability or individual acceptance alone; rather, teachers’ integration decisions are closely shaped by the alignment between digital tools, instructional tasks, classroom dynamics, and institutional contexts (20). Although previous studies have identified factors such as task–technology fit, psychological mechanisms, external support, self-efficacy, and privacy concerns as relevant to technology adoption (21, 22), research on physical education teachers’ adoption of smart wearable devices remains limited. This gap is particularly important because physical education is a movement-oriented and task-intensive teaching context in which smart wearables must support real-time monitoring, performance feedback, safety management, and data-informed instructional adjustment. Therefore, a more comprehensive theoretical framework is needed to explain the mechanisms and contextual conditions shaping teachers’ continuance intention.

Physical education teaching is a highly task-oriented instructional context in which teachers are required to demonstrate skills, monitor students’ movement performance, provide immediate feedback, and manage safety in real time (23). Because smart wearable devices are expected to support these concrete instructional tasks, the Task–Technology Fit (TTF) model is appropriate for explaining whether the technological functions of wearables align with the practical demands of Physical education teaching (16). However, teachers’ continuance intention cannot be explained by task–technology alignment alone. It also depends on whether teachers perceive the devices as useful, easy to use, and socially supported in school settings (106). These perceptual and social determinants are well captured by Unified Theory of Acceptance and Use of Technology (UTAUT). Therefore, integrating TTF and UTAUT provides a more comprehensive framework for understanding PE teachers’ continuance intention to use smart wearable devices.

Accordingly, this study examines K–12 physical education teachers’ continuance intention to use smart wearable devices in the context of school-based physical activity promotion. To provide a more comprehensive explanation, the study integrates task–technology fit and technology acceptance perspectives and further incorporates self-efficacy and perceived data privacy as extended factors. By doing so, this study aims to explain not only the net effects of individual determinants on teachers’ continuance intention, but also the multiple causal configurations through which technological, task-related, psychological, and contextual conditions jointly shape adoption pathways. Based on these objectives, the study addresses the following research questions:

RQ1: What factors influence K–12 physical education teachers’ continuance intention to use smart wearable devices in school-based physical activity promotion?

RQ2: How do task–technology fit and teachers’ cognitive, social, and psychological perceptions jointly explain their continuance intention to use smart wearable devices?

RQ3: What configurations of technological, task-related, psychological, and contextual conditions lead to high or low continuance intention among K–12 physical education teachers?

## 2 Literature review and research model

### 2.1 Smart wearable devices in physical education

**Figure 1:**
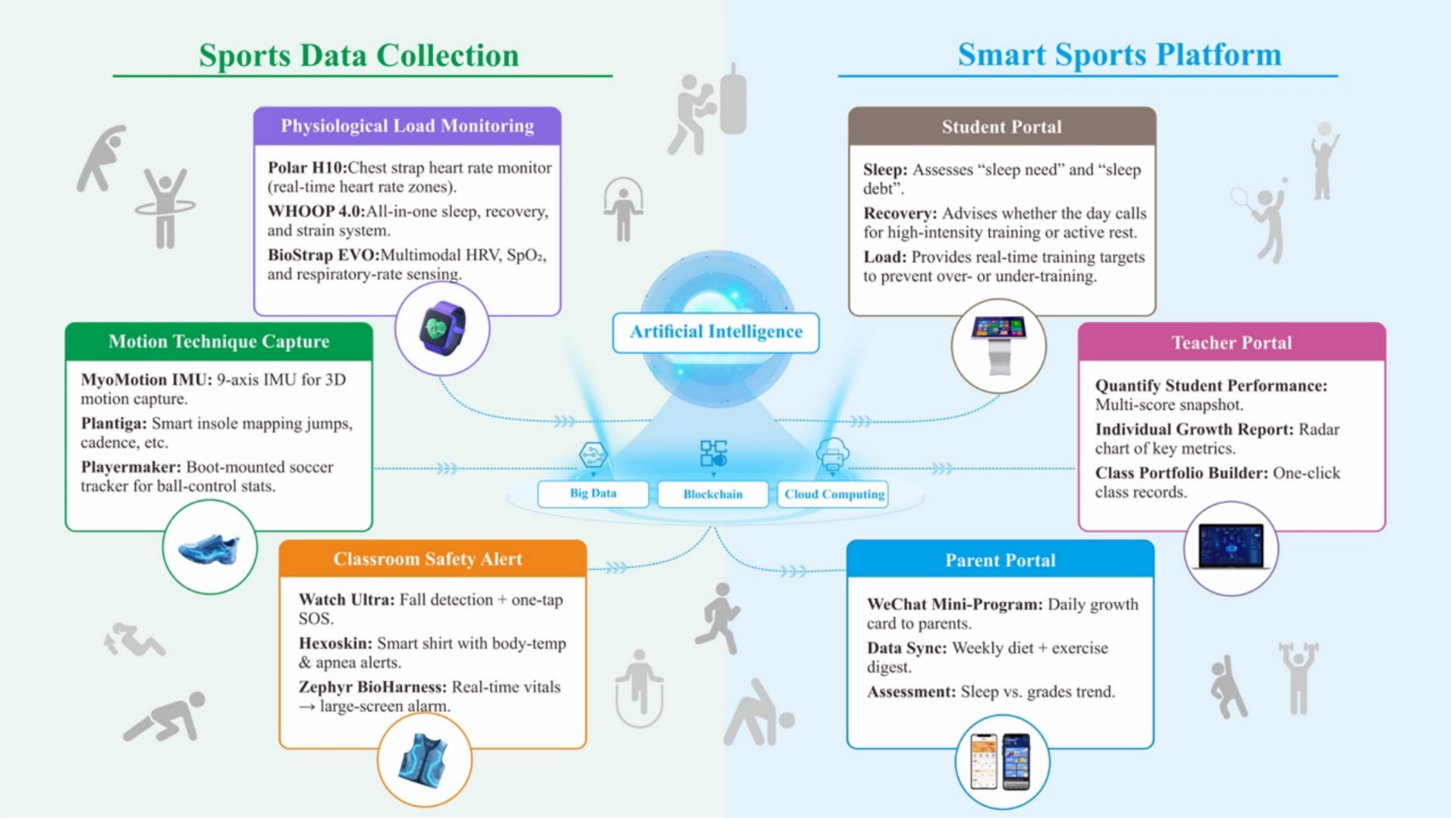
Smart wearable device system architecture.

Smart wearable devices, which integrate artificial intelligence with wearable technologies, have developed rapidly in recent years (24). Smartwatches and smart bracelets currently dominate the market, while smart glasses—an emerging category—are becoming a new driver of growth (25). These devices rely on sensor-based technologies, including (1) physiological monitoring tools (e.g., heart rate bands and myoelectric suits) used to track metabolic responses (26); (2) motion-tracking systems integrating GPS and inertial measurement units (IMUs) (27); and (3) multimodal devices combining environmental sensors with haptic feedback mechanisms (28). In terms of system architecture, smart wearable technologies typically consist of three layers: data acquisition, data transmission, and data processing (see Figure 1). Advances in multiplexed biosensors, microfluidic sampling technologies, and flexible materials have significantly improved the accuracy and wearability of smart wearable devices (29). The data acquisition layer collects learners’ real-time movement and physiological data (8), including step counts, physical activity levels, heart rate, and sleep quality, through devices such as smartwatches, heart-rate armbands, and smart glasses (30). The data transmission layer employs short-range wireless communication and networking technologies to synchronize device data with cloud platforms (15). The data processing layer uses cloud computing platforms and intelligent teaching systems to analyze large volumes of data and generate visual learning reports. Through interconnected platforms, teachers can access real-time dashboards displaying individual- and class-level data, providing valuable support for instructional monitoring and curriculum adjustment (19, 31).

In educational contexts, smart wearable devices provide three core capabilities: continuous biomechanical data acquisition (32), machine learning–based performance diagnostics (33), and predictive analytics for injury prevention (34). These capabilities are reflected in real-time movement correction through augmented feedback systems (35), personalized training programs based on biometric data (26), and evidence-based safety monitoring aimed at reducing training risks (36). A systematic review reported that wearable devices significantly improved body mass index (BMI) among children and adolescents, with a mean difference of −0.23 compared with controls (95% CI: −0.43 to −0.03; *p* = 0.03) (37). Wearable devices have emerged as a promising intervention for promoting physical activity among children and adolescents. Nearly half of the studies report positive effects (38), with greater benefits observed in guided-use contexts (39). Despite these advantages, recent studies have identified several challenges in the application of wearable technologies. For instance, device accuracy remains limited in certain exercise contexts (4), and prolonged use may reduce student motivation due to novelty fatigue (40). Additionally, device costs, data privacy concerns, and limited institutional capacity pose further barriers to the widespread adoption of smart wearables (41).

### 2.2 Task-technology fit model

The TTF model, originally proposed by Goodhue & Thompson (42), argues that technology use is more likely to produce favorable outcomes when technological functions fit the requirements of users’ tasks. Unlike acceptance-based models that mainly emphasize users’ perceptions and attitudes, TTF focuses on the structural alignment between task demands and technological capabilities. This perspective is particularly relevant to educational technology research because teachers’ technology use is embedded in specific instructional goals, classroom routines, and pedagogical tasks. Previous studies have applied TTF to various educational technology contexts, including learning management systems (43), VR–supported immersive learning (23), and visitor engagement in digital museums (44). Recent research further suggests that teachers’ digital integration cannot be fully explained by technology availability or individual acceptance alone; rather, it depends on how well digital tools align with instructional tasks and classroom contexts (20).

This task-oriented perspective is especially suitable for examining smart wearable use in physical education. Physical education is a movement-based, time-sensitive, and highly practical teaching context in which teachers must demonstrate motor skills, monitor students’ physical performance, provide immediate feedback, assess learning progress, and manage safety during class (45). Unlike many classroom subjects that rely mainly on verbal, textual, or screen-based interaction, physical education requires continuous coordination between teachers’ instructional decisions and students’ bodily performance (Li and Peng, 2026). In this context, technological characteristics refer to the functional capabilities of smart wearable devices, such as real-time data collection, physiological monitoring, motion tracking, automated feedback, and data visualization (46). Task characteristics refer to the instructional demands that physical education teachers need to address, such as curriculum delivery, skill monitoring, performance assessment, feedback provision, and safety supervision (47). Prior research on wearable technology adoption has shown that both task characteristics and technological characteristics can shape perceived task–technology fit, which in turn influences behavioral intention (48). However, limited research has examined how this mechanism operates in physical education. Therefore, this study proposes the following hypotheses:

*H*_1a_: TAC of smart wearable devices positively impacts task-technology fit.

*H*_1b_: TEC of smart wearable devices positively impacts task-technology fit.

*H*_2_: Task-technology fit of smart wearable devices positively impacts CBIU.

### 2.3 Unified theory of acceptance and use of technology model

The UTAUT was proposed by Venkatesh et al. (49), integrates several classical models—including the Technology Acceptance Model (TAM), Theory of Reasoned Action (TRA), Theory of Planned Behavior (TPB), and Diffusion of Innovations (DOI)—into a comprehensive framework for explaining technology adoption. The model identifies four core determinants of technology use: performance expectancy, effort expectancy, social influence, and facilitating conditions. By incorporating individual, social, and environmental factors, UTAUT has demonstrated strong explanatory power across educational technology contexts, including online teaching platforms (50), mobile learning systems (51), and other digital learning environments (52).

In the present study, UTAUT is used to explain the perceptual and social mechanisms underlying physical education teachers’ continuance intention to use smart wearable devices. Performance expectancy refers to the extent to which teachers believe that smart wearable devices can enhance instructional effectiveness and improve students’ physical performance (53). Effort expectancy refers to teachers’ perceptions of the ease of learning, operating, and integrating such devices into teaching practice (54). Social influence refers to the extent to which teachers perceive that important others—such as colleagues, school administrators, and students—expect or support their use of smart wearable devices (52). These constructs are especially relevant in physical education because smart wearable use occurs in fast-paced, movement-based teaching environments and often depends on school infrastructure, data management arrangements, and support from the professional community (54). In the original UTAUT framework, facilitating conditions are more directly related to actual use behavior than to behavioral intention, particularly in the earlier stages of technology adoption (51). Since this study focuses on continuance intention rather than objectively measured usage behavior, facilitating conditions were not included in the proposed model, consistent with prior intention-oriented technology adoption research (55). Therefore, the following hypotheses are proposed:

*H*_3a_: PE has a significant positive effect on physical education teachers’ CBIU to use smart wearable devices.

*H*_3b_: EE has a significant positive effect on physical education teachers’ CBIU to use smart wearable devices.

*H*_3c_: SI has a significant positive effect on physical education teachers’ CBIU to use smart wearable devices.

### 2.4 Integration and expansion of the TTF-UTAUT model

Compared with single-theory approaches, integrated models generally provide stronger explanatory and predictive power for technology adoption because they capture both functional and perceptual determinants of use behavior (107). In the present study, TTF and UTAUT are integrated because they address complementary aspects of physical education teachers’ continuance intention to use smart wearable devices. TTF explains the functional and structural question of whether wearable technologies fit the task requirements of physical education teaching, while UTAUT explains the cognitive and social question of whether teachers perceive these technologies as useful, manageable, and socially supported. Therefore, the integration is not simply a combination of two widely used models; rather, it links the “task–technology alignment” logic with the “teacher acceptance” logic.

This integration is particularly necessary in the physical education context. First, physical education teaching requires teachers to complete concrete instructional tasks within limited class time, including skill demonstration, movement monitoring, immediate feedback, performance assessment, and safety management (108). The effectiveness of smart wearable devices therefore depends heavily on whether their technological capabilities align with these instructional demands. This is the central concern of TTF. Second, even when wearable devices fit teaching tasks, teachers may not continue using them unless they believe the devices can improve teaching quality, are easy to operate, and are supported by colleagues and school administrators. These subjective evaluations are well captured by UTAUT, particularly through performance expectancy, effort expectancy, and social influence (Wallace et al., 2023). Integrating TTF and UTAUT thus enables this study to explain both why smart wearable devices are pedagogically relevant and why teachers are willing to continue using them (45).

Prior research also suggests meaningful relationships between the constructs of the two models. Technological characteristics may influence not only perceived task–technology fit, but also effort expectancy by reducing operational complexity and lowering use barriers (45, 55, 56). This relationship is especially relevant for smart wearable devices because such devices are often designed with lightweight architectures, simplified interfaces, and focused functions. These features may reduce teachers’ cognitive load and strengthen their perception that the devices are easy to learn, operate, and integrate into physical education teaching (57). Therefore, the following hypothesis is proposed:

*H*_4_: TEC of smart wearable devices has a significant positive effect on teachers’ EE.

To further enhance the explanatory power of the integrated framework, this study incorporates self-efficacy and perceived data privacy as extended variables. Rooted in Social Cognitive Theory (SCT) (58), self-efficacy refers to an individual’s belief in their ability to perform a specific task (59). In technology adoption research, self-efficacy is widely recognized as an important predictor of behavioral intention because it shapes users’ confidence in learning, operating, and applying new technologies (109). This construct is particularly relevant to smart wearable use in physical education because teachers must not only operate the devices but also interpret physiological and movement-related data and translate such information into instructional decisions (60). Prior studies have also shown that self-efficacy can strengthen adoption intention in MOOCs, mobile learning, and smart wearable contexts (61) (110). Therefore, the following hypothesis is proposed:

*H*_5a_: SE regarding the use of smart wearable devices has a significant positive effect on teachers’ CBIU.

Perceived data privacy is incorporated because smart wearable devices collect and process sensitive information related to students’ physical condition, movement performance, and health-related indicators. Privacy risk refers to users’ concerns about the potential misuse of personal information generated, stored, or transmitted through technological systems (62). Prior research has shown that privacy and data security concerns can reduce adoption intention, especially in contexts involving sensitive personal information, such as healthcare and wearable technologies (63–65). Although some consumer-focused studies report weaker effects of privacy concerns (66, 67), the educational context is different because teachers have ethical and professional responsibilities for protecting students’ data (Zhou et al., 2024). Accordingly, even when teachers perceive smart wearable devices as useful and manageable, concerns about data storage, school information governance, or third-party access may independently reduce their willingness to continue using them. Therefore, the following hypothesis is proposed:

*H*_5b_: PDP concerns of smart wearable devices have a significant negative effect on teachers’ CBIU.

**Figure 2:**
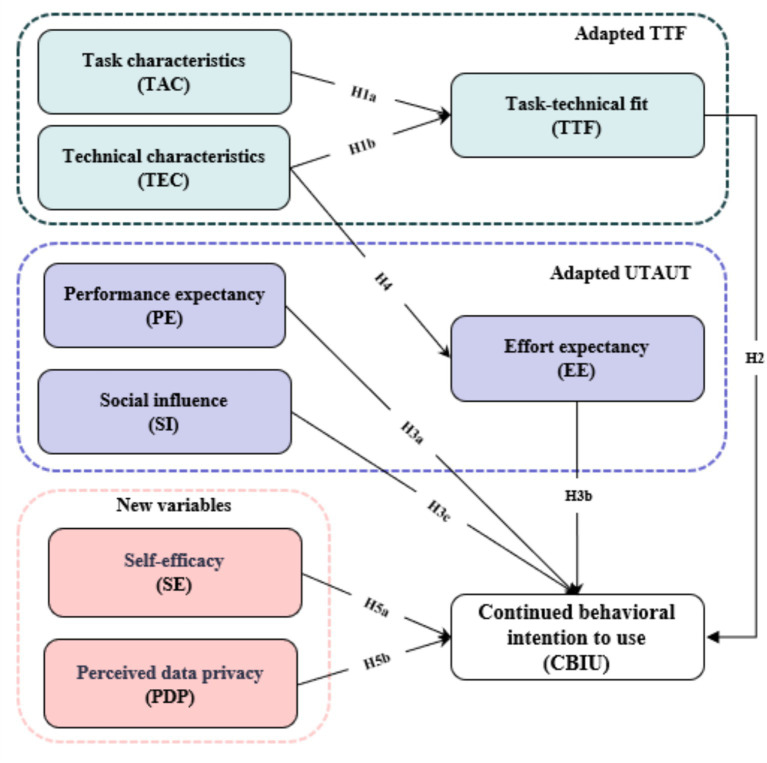
The research model proposed in our study.

In summary, based on the integration of the core constructs of the TTF and UTAUT models, this study introduces two additional psychological variables—self-efficacy and perceived privacy—to construct a research model that more comprehensively explains the mechanisms underlying physical education teachers’ adoption of smart wearable devices (see Figure 2). The proposed model is expected to provide both theoretical insights and empirical guidance for the practical integration of educational technology.

## 3 Research methodology

### 3.1 Sample and data collection

The project team distributed an electronic questionnaire during the 2025 National Symposium on “Digital Sport and Discipline Building” (From April 16th to 20th, 2025). The symposium focused on smart wearable technologies, addressing device functions, data analysis, application scenarios, and future development trends. During the training sessions, primary and secondary school physical education teachers actively engaged with smart wearable devices, quickly acquiring operational skills and gaining a deeper understanding of their applications in training, rehabilitation, and health management. The study was approved by the Research Ethics Committee of the Academic Committee of Henan Normal University (HNSD-2025-15-10), all operations and processes are carried out under the guidance and guidelines of the Declaration of Helsinki. Furthermore, the written informed consent of all participants was obtained in the questionnaire survey. If participants do not agree, they can stop answering at any time in wenjuanxing.^1^ A total of 755 valid questionnaires were collected, representing a sample size more than 10 times the number of items (27), thus satisfying the minimum sample size requirements for SEM (68, 69).

A total of 755 valid questionnaires were collected from K–12 physical education teachers. Because participants were recruited from a professional symposium focusing on digital sport and discipline building, this study employed a convenience sampling strategy rather than probability sampling. We acknowledge that participants self-selected into an event related to digital sport technologies; therefore, the sample may overrepresent teachers with relatively strong interest in educational technology or prior exposure to smart wearable devices, which may lead to higher continuance intention scores than would be observed in the broader K–12 physical education teacher population. The final sample included teachers from diverse demographic and school backgrounds: 500 respondents were male (66.22%) and 255 were female (33.78%); the largest age groups were 31–40 years old (*n* = 271, 35.89%) and 41–50 years old (*n* = 253, 33.51%); most respondents held a bachelor’s degree or below (*n* = 643, 85.17%); participants taught at primary schools (*n* = 364, 48.21%), junior high schools (*n* = 261, 34.57%), and high schools (*n* = 130, 17.22%); and they were from urban schools (*n* = 570, 75.50%), town schools (*n* = 74, 9.80%), and rural schools (*n* = 111, 14.70%). These characteristics provide useful contextual information for interpreting the findings, while the generalizability of the results should be considered in light of the non-probability sampling design. Relevant demographic information is presented in Table 1.

**Table 1:** Sample information (*N* = 755).

Demographics	Number	Percentage
	N	%
Gender		
Male	500	66.22
Female	255	33.78
Age		
20–30 years old	143	18.94
31–40 years old	271	35.89
41–50 years old	253	33.51
Over 50 years old	88	11.66
Educational background	643	85.17
Bachelor’s degree and below		
Master’s degree	110	14.60
Doctor’s degree	2	0.23
Educational stage		
Primary school	364	48.21
Junior high school	261	34.57
High school	130	17.22
Region		
Urban school	570	75.50
Town school	74	9.80
Rural school	111	14.70

### 3.2 Instruments

To investigate the adoption attitudes of primary and secondary school physical education teachers toward smart wearable devices, a questionnaire was developed using dimensions adapted from validated instruments in prior research. The questionnaire did not contain any sensitive personal information. The instrument comprised two sections. The first section gathered basic demographic data, including gender, age, educational background, teaching experience, and school location. The second section contained 27 items across nine dimensions, intended to assess respondents’ attitudes toward the adoption of smart wearable devices. All items were rated using a 5-point Likert scale ranging from 1 (strongly disagree) to 5 (strongly agree), enabling respondents to clearly express their levels of agreement.

To ensure the readability and accuracy of the questionnaire, the design process strictly followed a bilingual translation, back-translation, and pretesting procedure (70) in which the questionnaire was first translated from English to Chinese and then back-translated into English to ensure functional equivalence between the two language versions (46). A panel of 11 experts was invited to evaluate the questionnaire, including four physical education specialists, three educational technologists, two IT professionals, and two translation experts. They carefully reviewed, rephrased, and revised all questionnaire items to ensure clarity and appropriateness. A pilot survey was then conducted with 15 physical education teachers. Based on their feedback, the format, item sequence, and layout were adjusted to finalize the official version of the questionnaire (see Supplementary file).

### 3.3 Data analysis

The primary objective of this study was to investigate the factors influencing the adoption of smart wearable devices by primary and secondary school physical education teachers, using a combination of SEM and fsQCA to better capture the complexity of interrelationships among variables (71). SEM was employed to examine the net effects of individual antecedent variables on the outcome variable and to assess symmetrical relationships among variables. SPSS 26.0 software and AMOS 27.0 software were used to analyze the causal relationships underlying physical education teachers’ continuous usage intention, and to verify the individual effects of each antecedent variable (72). fsQCA, grounded in set theory, was applied to investigate how different configurations of conditions affect the outcome, particularly in handling asymmetric causal relationships. fsQCA software was used to identify and analyze the combinatorial effects of multiple causal conditions from a retrospective logic, aiming to determine the most parsimonious and sufficient explanations for the outcome. Thus, fsQCA was used to complement the SEM approach by uncovering alternative causal pathways (i.e., combinations of antecedent conditions) that lead to the outcome, thereby addressing the limitations of SEM in modeling causal asymmetry (73). The results obtained from both analytical methods are presented in detail in the following sections.

## 4 Results

### 4.1 Descriptive statistics and normality test of measurement items

Normality was assessed using skewness and kurtosis (74). Skewness reflects the symmetry of a distribution, whereas kurtosis reflects its peakedness (65). Since covariance-based SEM is sensitive to serious violations of normality, checking distributional properties is necessary before model estimation (75). Following the commonly accepted criteria of skewness < 3 and kurtosis < 10 (74), the data met the normality requirement.

As shown in Table 2, the mean values of the constructs ranged from 3.593 to 4.087, and the standard deviations ranged from 0.768 to 1.020. Skewness values ranged from −0.747 to −0.110, and kurtosis values ranged from −1.090 to 0.548, indicating no serious deviation from normality. Descriptively, task–technology fit (TTF, M = 4.083) and continued behavioral intention to use (CBIU, M = 4.087) showed the highest mean scores, suggesting that teachers generally perceived smart wearable devices as compatible with physical education teaching tasks and expressed a relatively strong intention to continue using them. By contrast, technological characteristics (TEC, M = 3.593) showed the lowest mean score, indicating comparatively less positive evaluations of current device functions. Perceived data privacy concerns (PDP, M = 3.777) were also evident, suggesting that privacy and data governance remain relevant concerns in the educational use of smart wearable.

**Table 2:** Results of descriptive statistics analysis.

Constructs	Indicator	Skewness	Kurtosis	Mean	Std. Dev.
TAC	TAC1	−0.343	−0.452	4.027	0.779
	TAC2	−0.316	−0.486		
	TAC3	−0.353	−0.466		
TEC	TEC1	−0.171	−1.002	3.593	1.020
	TEC2	−0.120	−1.016		
	TEC3	−0.110	−1.090		
TTF	TTF1	−0.682	0.527	4.083	0.797
	TTF2	−0.526	−0.012		
	TTF3	−0.611	0.245		
PE	PE1	−0.310	−0.506	3.947	0.809
	PE2	−0.167	−0.808		
	PE3	−0.373	−0.387		
EE	EE1	−0.723	0.548	4.043	0.844
	EE2	−0.747	0.637		
	EE3	−0.539	−0.020		
SI	SI1	−0.239	−0.699	3.940	0.802
	SI2	−0.231	−0.730		
	SI3	−0.217	−0.688		
SE	SE1	−0.174	−0.742	3.917	0.900
	SE2	−0.287	−0.415		
	SE3	−0.253	−0.546		
PDP	PDP1	−0.293	−0.556	3.777	0.913
	PDP2	−0.269	−0.538		
	PDP3	−0.259	−0.589		
CBIU	CBIU1	−0.442	−0.251	4.087	0.768
	CBIU2	−0.413	−0.344		
	CBIU3	−0.484	−0.053		

TAC, Task characteristics; TEC, Technical characteristics; TTF, Task-technology fit; PE, Performance expectancy; EE, Effort expectancy; SI, Social influence; FC, Facilitating conditions; BI, Behavioral intention; CBIU, Continued behavioral intention to use.

### 4.2 Structural equation model analysis

#### 4.2.1 Findings on the measurement model

This study evaluated the reliability and validity of the measurement model using standardized factor loadings, Cronbach’s alpha, composite reliability (CR), average variance extracted (AVE), and discriminant validity (76). Standardized factor loadings above 0.50 are generally considered acceptable. All factor loadings exceeded 0.80, indicating strong indicator reliability. Cronbach’s alpha, a widely used indicator of internal consistency, is recommended to exceed 0.70 (77). All Cronbach’s alpha values ranged from 0.928 to 0.979, well above the recommended threshold. Composite reliability (CR), which provides a more precise estimate of internal consistency than Cronbach’s alpha, ranged from 0.929 to 0.979 across all constructs, indicating strong internal consistency. Average variance extracted (AVE) reflects the proportion of variance captured by a construct relative to measurement error. An AVE value above 0.50 is generally considered evidence of acceptable convergent validity (78, 79). AVE values ranged from 0.814 to 0.939, indicating strong convergent validity across all latent constructs.

These results indicate that the measurement model was sufficiently robust to support subsequent structural analysis (see Table 3). The high factor loadings, Cronbach’s alpha values, CR values, and AVE values suggest that the observed items reliably represented their corresponding latent constructs. This is important for interpreting the structural paths reported in Table 4, because the estimated relationships are less likely to be distorted by measurement error. For example, the non-significant path from technological characteristics to task–technology fit should be interpreted in light of the strong reliability of the technological characteristics construct, suggesting that this result is unlikely to be caused by poor measurement quality.

**Table 3:** Factors loadings, t-values, Cronbach’s alpha, and composite reliability.

Constructs	Indicator	Loading	Cronbach’s *α*	CR	AVE
TAC	TAC1	0.946	0.960	0.961	0.891
	TAC2	0.961			
	TAC3	0.924			
TEC	TEC1	0.955	0.979	0.979	0.939
	TEC2	0.982			
	TEC3	0.970			
TTF	TTF1	0.929	0.958	0.958	0.883
	TTF2	0.947			
	TTF3	0.943			
PE	PE1	0.885	0.928	0.929	0.814
	PE2	0.933			
	PE3	0.887			
EE	EE1	0.949	0.945	0.946	0.855
	EE2	0.952			
	EE3	0.870			
SI	SI1	0.953	0.956	0.956	0.879
	SI2	0.938			
	SI3	0.922			
SE	SE1	0.953	0.934	0.948	0.859
	SE2	0.885			
	SE3	0.941			
PDP	PDP1	0.963	0.961	0.962	0.894
	PDP2	0.969			
	PDP3	0.903			
CBIU	CBIU1	0.907	0.962	0.932	0.819
	CBIU2	0.900			
	CBIU3	0.909			

SD, Standard deviation; Std., Standardized factor loadings; α, Cronbach’s alpha coefficient; CR, Composite reliability; TAC, Task characteristics; TEC, Technical characteristics; TTF, Task-technology fit; PE, Performance expectancy; EE, Effort expectancy; SI, Social influence; FC, Facilitating conditions; BI, Behavioral intention; UB, Use behavior.

**Table 4:** Summary of the hypothesis testing results (H1a–H5b).

Hypotheses	Paths	Standardized coefficients(β)	S. E.	C. R.	Results
*H_1a_*	TAC → TTF	0.916^***^	0.025	37.302	Accepted
*H_1b_*	TEC → TTF	−0.010	0.014	−0.713	Rejected
*H_2_*	TTF → CBIU	0.504^***^	0.018	28.265	Accepted
*H_3a_*	PE → CBIU	0.214^***^	0.016	13.632	Accepted
*H_3b_*	EE → CBIU	0.095^***^	0.014	6.817	Accepted
*H_3c_*	SI → CBIU	0.165^***^	0.015	10.973	Accepted
*H_4_*	TEC → EE	0.311^***^	0.029	10.876	Accepted
*H_5a_*	SE → CBIU	0.097^***^	0.015	6.642	Accepted
*H_5b_*	PDP → CBIU	−0.107^***^	0.014	−7.544	Accepted

S. E., Standard error; C. R., Critical ratio; TAC, Task characteristics; TEC, Technical characteristics; TTF, Task-technology fit; PE, Performance expectancy; EE, Effort expectancy; SI, Social influence; SE, Self-efficacy; PDP, Perceived data privacy; CBIU, Continued behavioral intention to use.

Discriminant validity refers to the extent to which a latent construct is distinct from other constructs, reflecting its unique characteristics or conceptual meaning. Discriminant validity was assessed using the Fornell–Larcker criterion, which compares the square root of each construct’s AVE with its correlations with other constructs (79, 80). As shown in Table 5, the square root of the AVE for each construct exceeded its correlations with all other constructs, indicating adequate discriminant validity across the nine constructs. This result suggests that the constructs captured conceptually and empirically distinct dimensions, thereby reducing concerns about construct redundancy and supporting the validity of the proposed model.

**Table 5:** The values of convergent and discriminant validity.

Constructs	TAC	TEC	TTF	PE	EE	SI	SE	PDP	CBIU
TAC	**0.944**								
TEC	0.528	**0.969**							
TTF	0.858	0.855	**0.940**						
PE	0.795	0.643	0.731	**0.902**					
EE	0.747	0.385	0.731	0.635	**0.925**				
SI	0.750	0.729	0.679	0.790	0.622	**0.937**			
SE	0.787	0.702	0.702	0.856	0.629	0.858	**0.927**		
PDP	0.669	0.801	0.579	0.777	0.523	0.806	0.838	**0.946**	
CBIU	0.885	0.506	0.860	0.798	0.766	0.756	0.776	0.636	**0.905**

Square roots of AVE values are shown in bold on diagonals. TAC, Task characteristics; TEC, Technical characteristics; TTF, Task-technology fit; PE, Performance expectancy; EE, Effort expectancy; SI, Social influence; SE, Self-efficacy; PDP, Perceived data privacy; CBIU, Continued behavioral intention to use.

The measurement models were evaluated using maximum likelihood estimation (MLE), and the results indicated good overall model fit. Table 6 presents the model fit indices for the three models. For the research model, the chi-square value was χ^2^ = 1124.492 with 292 degrees of freedom (*p* < 0.001). Although the chi-square test was statistically significant, its sensitivity to sample size requires that model fit be evaluated in conjunction with other indices. The chi-square to degrees of freedom ratio (χ^2^/df) was 3.851, falling within the acceptable range of 2 to 5 and indicating good model fit (81, 82). The root mean square error of approximation (RMSEA) was 0.058, below the 0.08 threshold, indicating acceptable model fit (76), The Comparative Fit Index (CFI) and Tucker–Lewis Index (TLI) were 0.982 and 0.977, respectively, both exceeding the 0.95 benchmark and indicating excellent model fit (76). The standardized root mean square residual (SRMR) was 0.021, well below the 0.08 threshold, further supporting good model fit. Taken together, these indices indicate that the measurement model fits the data well, demonstrates strong validity and reliability, and supports the proposed hypotheses and theoretical framework.

**Table 6:** The goodness-of fit indices of the measurement models.

Goodness-of-fit-indices	χ^2^/df	GFI	TLI	CFI	SRMR	RMSEA
Criteria	<5	>0.9	>0.9	>0.9	<0.08	<0.08
Adapted TTF	4.925	0.936	0.946	0.958	0.033	0.071
Adapted UTAUT	4.353	0.942	0.963	0.969	0.026	0.066
Research mode	3.851	0.957	0.977	0.982	0.021	0.058

#### 4.2.2 Findings on the structural model

Path coefficients and their statistical significance were used to test the proposed hypotheses (see Table 4). This approach allows the strength and direction of the relationships among latent constructs in the integrated TTF–UTAUT framework to be directly assessed, and provides evidence for explaining the theoretical mechanisms underlying physical education teachers’ continuance intention to use smart wearable devices. The results indicate that TAC has a significant positive effect on TTF (*β* = 0.916, *p* < 0.001), supporting *H_1a_*. In contrast, TEC has no significant effect on TTF (*β* = −0.010, *p* > 0.05), indicating that *H_1b_* is not supported. This finding aligns with current trends in educational technology, which emphasize that the effectiveness of technology integration depends more on its alignment with instructional tasks than on technological complexity alone (83).

The effect of TTF on users’ CBIU is significant (*β* = 0.504, *p* < 0.001), supporting Hypothesis *H_2_*. This indicates that physical education teachers are more likely to sustain their use of smart wearable devices when the technology closely aligns with the functional requirements of teaching and learning tasks in physical education. This finding is consistent with prior research on smart wearables in physical education (31), which highlights the pivotal role of TTF in shaping behavioral intention. However, other studies report no statistically significant effect (55, 84). These discrepancies may be attributed to differences in research contexts, sample characteristics, or specific application scenarios of the technology.

The continuous usage intention of physical education teachers is significantly and positively influenced by PE (*β* = 0.214, *p* < 0.001), EE (*β* = 0.095, *p* < 0.01), and SI (*β* = 0.165, *p* < 0.01), supporting *H_3a_*, *H_3b_*, and *H_3c_*. These findings are consistent with prior UTAUT-based studies (45, 85, 86) and indicate that the effect of PE exceeds that of SI and EE (87). When physical education teachers perceive that smart wearable devices enhance teaching effectiveness, support exercise monitoring, facilitate health management, and are simple and intuitive to use, recommendations from peers, school administrators, and professional organizations play a significant role in shaping their adoption decisions. This, in turn, strengthens their intention to use these devices consistently.

The results also indicate that TEC has a significant positive effect on EE (*β* = 0.311, *p* < 0.001), supporting *H_4_*. This finding is consistent with prior research (31). The ease of use, stability, and functional design of smart wearable devices directly influence teachers’ perceived ease of use. In various educational contexts, optimizing technical features—such as lightweight design, extended battery life, and seamless data synchronization—reduces usability barriers and indirectly enhances teachers’ intention to use the devices consistently.

SE has a significant positive effect on CBIU (*β* = 0.097, *p* < 0.01), whereas PDP has a significant negative effect (*β* = −0.107, *p* < 0.01), supporting Hypotheses *H_5a_* and *H_5b_*. Teachers’ self-efficacy is a critical competency in addressing challenges associated with emerging technologies (88). In the context of physical education, teachers’ confidence in their ability to use technology (i.e., self-efficacy) directly influences their intention to adopt it. This relationship can be explained by social cognitive theory (89). Teachers with high self-efficacy tend to demonstrate greater adaptability to technology. They not only master basic operations quickly but also actively explore advanced features. This positive orientation translates into a stronger intention to continue using the devices. Numerous studies indicate that privacy concerns negatively affect users’ acceptance of technology (63, 64, 90), although some consumer-focused studies report contrasting results (66). The results indicate that physical education teachers’ perceptions of privacy risks reduce their intention to use smart wearable devices. Teachers evaluate both perceived threats and coping mechanisms; when privacy safeguards are insufficient, their sense of professional responsibility reduces their intention to adopt the devices (91). In physical education settings, data security and privacy protection are key factors influencing teachers’ continuous usage intentions (92).

**Figure 3:**
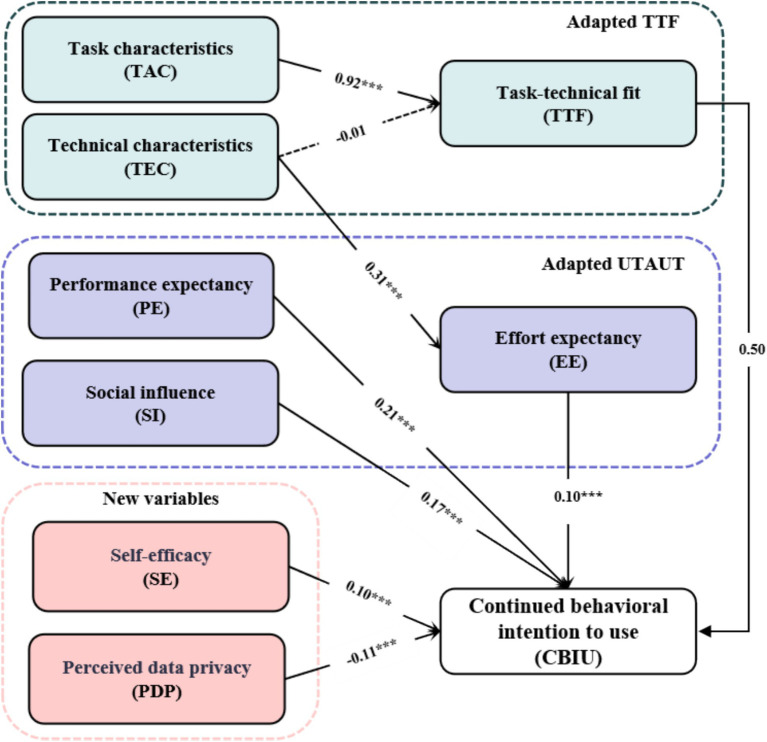
Hypothesis testing results.

Beyond statistical significance, the practical importance of the predictors was further interpreted using Cohen’s (93) guidelines for standardized coefficients, where values around 0.10, 0.30, and 0.50 indicate small, medium, and large effects, respectively. Task–technology fit showed the largest effect on CBIU (*β* = 0.504), indicating that a one-standard-deviation increase in task–technology fit was associated with a 0.504-standard-deviation increase in continuance intention, equivalent to approximately 0.39 points on the five-point CBIU scale given its observed standard deviation of 0.768. PE (*β* = 0.214) and SI (*β* = 0.165) showed smaller but meaningful effects, while SE (*β* = 0.097) and EE (*β* = 0.095) had relatively small effects. PDP had a significant but small negative effect (*β* = −0.107), suggesting that privacy concern’s function more as an inhibiting factor than as a dominant barrier. Overall, these results indicate that improving the alignment between smart wearable functions and physical education teaching tasks may yield greater practical benefits than focusing only on ease of use, social pressure, or privacy concerns. All hypotheses were statistically supported except for the relationship between technological characteristics and task–technology fit. A schematic representation of the path coefficient analysis is presented in Figure 3.

### 4.3 Fuzzy-set qualitative comparative analysis

#### 4.3.1 Calibration

In this study, eight variables—TAC, TEC, TTF, PE, EE, SI, SE, and PDP—are specified as antecedent conditions, while teachers’ continuous usage intention serves as the outcome variable. Calibration required calculating the mean values of each variable. Subsequently, the calibrate() function in fsQCA 4.0 was used to transform the raw data into values ranging from 0 to 1, based on three calibration thresholds (5, 50, and 95%), representing full membership, the crossover point, and full non-membership, respectively (94, 95).

#### 4.3.2 Necessary condition analysis

To conduct the necessity analysis, two criteria—consistency and coverage—are evaluated (See Table 7). A consistency value above 0.90 indicates a necessary condition, while a coverage value above 0.80 is considered sufficient (96). For both high and low levels of continued behavioral intention to use, none of the antecedent conditions reached the consistency threshold required to be considered necessary. This result indicates that teachers’ continuance intention is not driven by any single condition alone. Instead, it is more likely to emerge from combinations of task-related, technological, psychological, and contextual factors. The absence of necessary conditions therefore supports the use of fsQCA as a complementary method to SEM and justifies the subsequent configurational analysis of multiple sufficient pathways.

**Table 7:** Analysis of necessary conditions.

Antecedent conditions	CBIU		~CBIU	
	Consistency	Coverage	Consistency	Coverage
TAC(~TAC)	0.896 (0.497)	0.780 (0.601)	0.646 (0.775)	0.545 (0.778)
TEC(~TEC)	0.676 (0.596)	0.682 (0.632)	0.628 (0.662)	0.592 (0.656)
TTF(~TTF)	0.811 (0.567)	0.797 (0.581)	0.561 (0.779)	0.548 (0.782)
PE(~PE)	0.789 (0.544)	0.791 (0.582)	0.581 (0.776)	0.543 (0.775)
EE(~EE)	0.800 (0.569)	0.729 (0.589)	0.575 (0.798)	0.555 (0.793)
SI(~SI)	0.871 (0.520)	0.783 (0.633)	0.677 (0.741)	0.568 (0.789)
SE(~SE)	0.813 (0.544)	0.795 (0.598)	0.608 (0.775)	0.555 (0.795)
PDP(~PDP)	0.706 (0.607)	0.748 (0.614)	0.591 (0.745)	0.584 (0.703)

“~” denotes “negation”.

#### 4.3.3 Configuration adequacy analysis

Since no single antecedent condition is identified as necessary for the outcome, a configurational analysis is conducted. A truth table with 2^k^ rows is constructed, where k denotes the number of antecedent conditions and each row represents a possible configuration. In the QCA analysis, the consistency threshold is set at 0.80, and the PRI (proportional reduction in inconsistency) threshold is set at 0.70 (97). According to Fiss (73), a minimum frequency threshold of 3 is appropriate for large samples (n > 150). Therefore, a frequency threshold of 3 is adopted to ensure the robustness of the configurational analysis. The truth table is then minimized to identify configurations of antecedent conditions associated with physical education teachers’ continuous usage intention of smart wearable devices. Both intermediate and parsimonious solutions are used. An antecedent condition appearing in both solutions is classified as a core condition, indicating a strong causal relationship with the outcome. Otherwise, it is classified as a peripheral (marginal) condition. The configurational analysis identifies four pathways leading to high continuous usage intention and five pathways leading to low continuous usage intention (see Table 8). The overall consistency of the high and low continuous usage intention configurations is 0.955 and 0.945, respectively, and the overall coverage is 0.647 and 0.567 indicating that the configurations had strong explanatory adequacy. These results suggest that teachers’ continuance intention is not a single, homogeneous phenomenon, but emerges from different combinations of task-related, technological, psychological, and contextual conditions. Understanding these distinct patterns is important for designing targeted interventions, because different adoption pathways may require different support strategies rather than a uniform approach.

**Table 8:** Intermediate solution results.

Variable	High CBIU				Non-high CBIU				
	M1a	M1b	M2a	M2b	N1a	N1b	N2a	N2b	N3
TAC	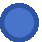	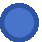	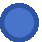	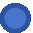		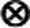	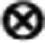	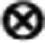	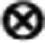
TEC	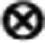	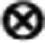	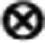		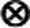	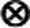	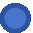	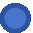	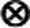
TTF	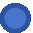	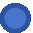	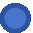		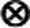		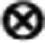	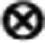	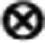
PE	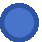		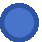	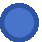	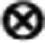	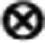	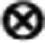	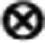	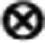
EE	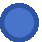	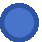		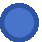	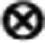	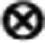	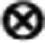	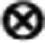	
SI		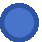	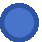	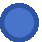	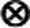	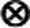		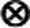	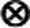
SE		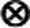	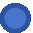	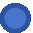	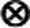	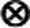	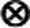		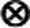
PDP	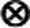	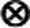	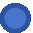	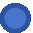	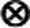	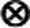	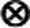	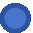	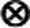
Consistency	0.981	0.971	0.975	0.968	0.965	0.971	0.956	0.968	0.972
Raw coverage	0.356	0.308	0.353	0.527	0.405	0.392	0.310	0.287	0.405
Unique coverage	0.025	0.030	0.013	0.018	0.024	0.010	0.039	0.040	0.023
Solution consistency	0.955				0.945				
Solution coverage	0.647				0.567				

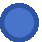
 indicate that the condition exists, 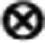 indicate that the condition is missing, and blank Spaces indicate that the condition is irrelevant. Large ICONS indicate that the conditions are core conditions, while small ICONS indicate that the conditions are edge conditions.

The four pathways leading to high continuous usage intention are classified into two categories: (1) task motivation (M1), characterized by task characteristics and effort expectancy, and (2) performance support (M2), characterized by performance expectancy and social influence.

Configuration M1 comprises two pathways, both characterized by task characteristics and effort expectancy as core conditions, with technological characteristics as a peripheral condition. This configuration can be interpreted as a task-motivation pattern, in which teachers’ continuance intention is mainly sustained by perceived task value and willingness to invest effort. Path M1a is characterized by high task characteristics, high performance expectancy, high effort expectancy, and low technological characteristics. This pathway suggests that teachers may continue using smart wearable devices when they believe these devices are useful for teaching tasks and can improve instructional performance, even if they perceive the devices as technically limited. In other words, these teachers appear to prioritize pedagogical applicability over technological sophistication. When smart wearable devices are perceived as capable of supporting key teaching tasks, such as monitoring, feedback, and performance assessment, even relatively basic devices may sustain teachers’ continuance intention through strong task motivation (98). Path M1b is characterized by high task characteristics, high effort expectancy, strong social influence, and low technological characteristics. This pathway also reflects a task-motivation pattern, but with a different supporting mechanism. Teachers in this pathway perceive the devices as relevant to their teaching tasks and are willing to invest effort in learning and using them; however, strong social support helps compensate for perceived technological limitations. In this sense, social influence functions as an external enabling condition that reinforces teachers’ willingness to continue use. If such support from colleagues, administrators, or the school environment is weakened, teachers’ continuance intention may become less stable, even when they personally recognize the task value of the technology. In this sense, social influence functions as an external enabling condition that reinforces teachers’ willingness to continue use. If such support from colleagues, administrators, or the school environment is weakened, teachers’ continuance intention may become less stable, even when they personally recognize the task value of the technology. Cui et al. (99) suggested that when technological features do not fully meet users’ expectations, continued use may depend on two mechanisms: whether users believe they can learn the technology and whether they feel supported by their organization.

Configuration M2 comprises two pathways, with performance expectancy and social influence as core conditions. This configuration can be interpreted as a performance-support pattern, in which teachers’ continuance intention is mainly sustained by perceived instructional benefits and support from the surrounding professional environment. Path M2a is characterized by high task characteristics, strong performance expectancy, strong social influence, and low technological characteristics. This pathway suggests that teachers may continue using smart wearable devices when they perceive clear instructional benefits and receive support from colleagues, administrators, or the school environment, even if the devices are technically limited. In this case, institutional support and perceived effectiveness can buffer the negative influence of weak technological characteristics, consistent with health-related wearable adoption studies (48).

Path M2b is characterized by high performance expectancy, high effort expectancy, and strong social influence, with task characteristics absent. This configuration leads to high continuous usage intention, even when teachers perceive the device as less important for their teaching tasks (100).

In examining physical education teachers’ low continuous usage intention toward wearable devices, three distinct configurations (N1, N2, N3) and their corresponding sub-configurations are identified based on the dominant core conditions.

Configuration N1 is characterized by low performance expectancy and low effort expectancy as core conditions. This suggests that teachers in this configuration have limited expectations regarding the ability of smart wearable devices to improve teaching performance and are unwilling to invest substantial effort in learning or using them, leading to low continuance intention (101). Its sub-configurations, N1a and N1b, include peripheral conditions such as low technological characteristics, poor task–technology fit, low self-efficacy, weak social influence, and low perceived data privacy concerns, which further reinforce low continuance intention. Poor task–technology fit confirms teachers’ skepticism about the instructional value of the devices, low self-efficacy weakens their motivation to try, and weak social influence means that external support is insufficient to offset their withdrawal tendency. Notably, low perceived data privacy concerns in this configuration do not promote adoption; rather, these teachers may be relatively indifferent to privacy issues because they have already decided not to use the technology.

Configuration N2 is characterized by low task characteristics, low task–technology fit, low performance expectancy, and low effort expectancy as core conditions, with multiple unfavorable factors jointly contributing to low continuous usage intention (102). Its two sub-configurations differ in their peripheral conditions. N2a is characterized by high technological characteristics, low self-efficacy, and low perceived data privacy concerns. This pathway describes teachers who face relatively advanced or complex devices but lack confidence in their ability to use them effectively. N2b is characterized by high technological characteristics, high perceived data privacy concerns, and low social influence. This pathway describes teachers who may recognize the technological functions of the devices but remain concerned about data protection and receive insufficient support from their school or professional environment. Together, these pathways suggest that technological sophistication alone is insufficient to sustain adoption when task relevance, perceived usefulness, ease of use, and support conditions are weak.

Configuration N3 is characterized by low task characteristics, low task–technology fit, and low performance expectancy as core conditions. Teachers perceive wearable devices as having limited importance for teaching tasks, poor compatibility with existing teaching environments and technological infrastructure, and low potential to improve instructional performance, all of which jointly result in low continuous usage intention (103).

These configurations demonstrate that physical education teachers’ continuous usage intention toward wearable devices is shaped by the interplay of multiple factors. High continuous usage intention is primarily associated with teachers’ positive perceptions of the devices’ importance for teaching tasks, potential for performance enhancement, social support, and effort expectancy. Conversely, low continuous usage intention stems from negative perceptions of these core conditions combined with deficiencies in peripheral conditions. These findings provide a more comprehensive perspective for understanding physical education teachers’ usage behavior regarding wearable devices.

## 5 Discussion

### 5.1 Theoretical implications

This study extends existing technology adoption theories by applying an integrated TTF–UTAUT framework to the context of smart wearable use in physical education. The findings show that physical education teachers’ continuance intention is shaped by both task–technology alignment and teachers’ cognitive and social evaluations of the technology. Compared with single-theory approaches, the integrated framework provides a more comprehensive explanation because it captures both the functional fit between wearable devices and teaching tasks and the perceptual mechanisms through which teachers evaluate usefulness, ease of use, and social support.

First, the results highlight the central role of task–technology fit in explaining continuance intention. While UTAUT constructs such as performance expectancy, effort expectancy, and social influence explain teachers’ subjective perceptions, they do not fully explain how technological attributes are translated into sustained use (111). The inclusion of TTF addresses this limitation by showing that task characteristics and technological characteristics shape teachers’ perceived fit, which in turn strongly predicts continuance intention (Kim et al., 2025) In this study, TTF exerted the strongest effect on continued behavioral intention to use, suggesting that physical education teachers prioritize whether smart wearable devices can support concrete instructional tasks over whether the devices are technologically advanced. This finding reflects the distinctive structure of physical education teaching. Unlike classroom subjects in which technology often serves as a medium for information delivery, physical education requires real-time coordination between teaching decisions and students’ bodily performance. If a wearable device cannot provide timely heart-rate alerts, movement feedback, or performance information during a lesson, it is not merely “less useful”; it may become pedagogically impractical. Thus, in physical education, task–technology fit functions as a prerequisite for forming positive performance expectancy and effort expectancy.

Second, the inclusion of self-efficacy and perceived data privacy expands the explanatory scope of UTAUT in educational settings involving sensitive student data. Self-efficacy directly influenced teachers’ continuance intention, confirming that teachers’ confidence in operating wearable devices and interpreting physiological or movement-related data plays a role in sustained use (112). However, its relatively small effect may reflect the comparatively low operational complexity of many consumer-grade wearable devices, such as smart bracelets and heart-rate monitors (48). Once teachers overcome the initial setup stage, self-efficacy may become less decisive than task fit and perceived instructional value. Perceived data privacy, by contrast, had a significant negative effect, indicating that concerns about student health data, movement records, and third-party access can inhibit continued use (104). In school contexts, privacy concerns extend beyond personal risk because teachers also carry professional and ethical responsibilities for protecting students’ data. This finding extends UTAUT by incorporating a risk-related factor that is highly relevant to smart wearable use in physical education.

Third, the combined use of SEM and fsQCA strengthens the analytical contribution of this study. SEM identified the net linear effects of individual predictors, confirming that TTF was the strongest predictor of continuance intention. fsQCA further revealed that teachers’ continuance intention emerges from multiple configurations rather than from a single determinant. The two approaches therefore provide complementary evidence. For example, SEM showed that technological characteristics did not directly predict task–technology fit, whereas fsQCA showed that technological characteristics still appeared as a peripheral condition in several configurations. This is not contradictory; rather, it indicates that technological characteristics may not independently determine perceived fit, but can still facilitate or constrain adoption under specific combinations of task, psychological, and social conditions. Together, SEM and fsQCA reveal both the linear relationships and the causal complexity underlying physical education teachers’ continuance intention (71).

### 5.2 Practical implications

The findings offer practical implications for policy, teacher development, and technology design. First, at the policy level, educational authorities should move beyond a technology-supply orientation and prioritize task-aligned implementation. The non-significant effect of technological characteristics on task–technology fit suggests that more advanced hardware does not automatically produce greater teaching value. Procurement and evaluation standards should therefore require vendors to demonstrate how devices directly support physical education learning objectives, such as real-time heart-rate zone alerts for moderate-to-vigorous physical activity targets, automated step counting for fitness assessment, and feedback functions for movement correction. In addition, policies should establish clear standards for device access, data storage, privacy protection, and third-party data use (Wu et al., 2026). Professional development systems should also incorporate smart wearable use into the digital competence framework for physical education teachers (Zhang et al., 2026).

Second, at the teacher-development level, schools should provide differentiated support rather than relying only on uniform digital literacy training. For task-motivated teachers, training should focus on task–technology mapping, such as using heart-rate data to adjust activity intensity or using movement feedback to support skill correction (Ibrahim et al., 2026). For teachers who recognize the value of wearable devices but struggle with implementation, peer mentoring and department-level communities of practice may be more effective than one-time workshops. School administrators can designate experienced early adopters as technical mentors to provide continuous, context-specific support (105). For teachers with general optimism toward technology but weak task awareness, professional development should help translate positive attitudes into concrete teaching routines. Schools should also provide stable infrastructure, device maintenance, data dashboards, technical assistance, and clear privacy protocols. These measures can reduce implementation burden and help teachers integrate smart wearables into existing physical education routines rather than treating them as additional work (Li and Peng, 2026).

Third, at the technology-design level, developers should treat teachers’ classroom feedback as a key basis for device improvement (Žerovnik, 2024). Smart wearable devices should be compatible, scalable, and easy to integrate into existing physical education systems (Baig & Yadegaridehkordi, 2025). A gradual integration strategy is recommended: schools can begin with simple functions such as heart-rate monitoring, activity tracking, and class-level feedback, and then move toward more advanced applications such as individualized performance analysis and data-informed curriculum adjustment. This stepwise approach can help teachers build confidence and reduce resistance. Developers should also improve algorithms, interfaces, and data visualization tools to reduce the time required for device configuration, data management, and interpretation (31). To address privacy concerns, developers and schools should provide user-friendly privacy controls, transparent explanations of data rights, and regular reports on data usage (Zhao & Su, 2026).

Taken together, the sustainable integration of smart wearable devices into physical education requires coordination among policy support, teacher empowerment, and technology optimization. Technology design should not be viewed only as product improvement, but as part of a broader educational ecosystem in which institutional support, teacher needs, and privacy governance jointly shape continued use (Giugliano et al., 2026).

### 5.3 Limitations and future research directions

This study provides valuable insights into the mechanisms underlying physical education teachers’ continuance intention to use smart wearable devices; however, several limitations should be acknowledged. First, the use of a convenience sample recruited from a technology-focused professional symposium may have overrepresented teachers who already held positive attitudes toward educational technology or had prior exposure to smart wearable devices. This may have inflated the average level of continued behavioral intention to use and reduced the variability of key predictors. In addition, the Chinese educational context may shape the meaning of constructs such as social influence and performance expectancy in ways that may not fully apply to more decentralized educational systems, such as those in the United States or other countries (87). Second, this study focused primarily on commercial smart wearable devices, with limited attention to open-source or education-initiated customized platforms. Different platform types may involve different levels of flexibility, data governance, and pedagogical adaptability. Third, the study relied on self-reported cross-sectional data, which limits causal inference. Common method bias may also have inflated the observed relationships among perceptual variables, such as task–technology fit, performance expectancy, and effort expectancy, because they were measured using the same questionnaire at the same time point. Although Harman’s single-factor test did not indicate serious bias, this method has known limitations and cannot fully rule out common method variance.

Future research could address these limitations in several ways. First, future studies should use stratified or probability-based sampling across regions, school types, and educational levels to improve representativeness and enhance the generalizability of findings. Second, researchers could compare commercial smart wearable platforms with open-source or school-developed systems to examine how different technological ecosystems influence teachers’ sustained use and instructional integration (Khan et al., 2025). Third, longitudinal research should track teachers from initial adoption to sustained use over 12 to 24 months to establish causal direction and determine whether the configurational pathways identified in this study predict actual continued behavior rather than temporary intention. Future studies should also incorporate objective behavioral data, such as device usage logs, classroom observations, and platform records. Finally, comparative cross-cultural studies are needed to examine whether the task–technology fit-dominant pattern found in this study also exists in decentralized educational systems, where social influence may operate more through voluntary peer collaboration than through administrative expectations or institutional compliance.

## 6 Conclusion

This study investigated K–12 physical education teachers’ continuance intention to use smart wearable devices by integrating the UUF model and the UTAUT, with SE and PDP incorporated as extended variables. Using both SEM and fsQCA, the study examined not only the net effects of individual predictors but also the configurational pathways leading to different levels of continuance intention.

The SEM results showed that task–technology fit was the strongest predictor of teachers’ continuance intention, followed by PE, SI, SE, and EE, whereas PDP had a negative effect. These findings indicate that physical education teachers are more likely to continue using smart wearable devices when the devices are perceived as well aligned with teaching tasks, useful for improving instructional performance, easy to use, and supported by the surrounding professional environment. The fsQCA results further revealed multiple pathways leading to high and low continuance intention, confirming that continued use does not depend on a single determinant but emerges from different combinations of task-related, technological, psychological, and contextual conditions.

Overall, this study contributes to the literature by demonstrating the value of combining symmetric and asymmetric analytical approaches to explain technology adoption in physical education. The findings highlight the central importance of aligning smart wearable functions with the practical demands of physical education teaching, while also emphasizing the roles of teacher perceptions, social support, self-efficacy, and privacy concerns. These results provide theoretical and practical guidance for policymakers, school administrators, and technology developers seeking to promote the sustainable integration of smart wearable devices into physical education and school-based physical activity promotion.

## Data Availability

The raw data supporting the conclusions of this article will be made available by the authors, without undue reservation.

## Footnotes

1
https://www.wjx.cn/
