# Supplementary material for: Smart wearables for school-based physical activity promotion: K–12 physical education teachers’ continuance intention and adoption pathways
Source: Front Public Health. 2026 Jun 18;14:1756723. doi: 10.3389/fpubh.2026.1756723 (PMC13323152; doi:10.3389/fpubh.2026.1756723)
Supplement: Supplementary file 1 [file Table_1.DOCX]

**Supplementary file: Questionnaire and Measurement Items**

A Questionnaire on the Use of Smart Wearable Devices by Physical Education Teachers

Dear Teachers,

Greetings!

We sincerely appreciate your willingness to take the time to complete this questionnaire. The purpose of this survey is to understand how physical education teachers use smart wearable devices in their teaching practice.

Participation in this questionnaire is entirely voluntary and completely anonymous. It is intended solely for academic research and does not involve any evaluation of your personal teaching performance. We assure you that your participation will pose no risk to your personal well-being. All data collected will be used strictly for scholarly purposes and will remain confidential. Please respond truthfully based on your actual teaching experience. There are no “right” or “wrong” answers — simply choose the option that best reflects your first instinct.

If you feel any discomfort while completing the survey, you are free to withdraw at any time, and your responses will not be recorded. By completing and submitting the questionnaire, we will consider that you have given your informed consent.

Thank you once again for your thoughtful and honest participation.

**Part 1. Demographic Information**

**A. Gender**: .

1. Male 2. Female

**B. Age range:**

Which of the following best describes your age group? **.**

1. 20-30 years old 2. 31-40 years old 3. 41-50 years old 4. Over 50 years old

**C.** **Highest educational qualification： .**

1. Bachelor’s degree and below 2. Master’s degree 3. Doctoral degree

**D. Your current teaching level: ________.**

1. Primary school 2. Junior high school 3. High School

**E. What is the regional location of your school?________.**

1. Urban school 2. Town school 3. Rural school

**Part 2. Main research scale**

| **Construct** | **Code** | **Items** | **Sources** |
| --- | --- | --- | --- |
| Task characteristics (TAC) | TAC1 | Using smart wearable devices does not significantly increase teaching complexity.  使用智能可穿戴设备不会显著增加教学复杂性 | (Zhou et al., 2010; Wang et al., 2020) |
|  | TAC2 | Smart wearable devices effectively support key teaching tasks like assessment and feedback.  智能可穿戴设备能够有效支持评估与反馈等关键教学任务 |  |
|  | TAC3 | Smart wearable devices help me monitor student performance and adjust instruction in real time.  智能可穿戴设备帮助我监测学生表现，并实时调整教学 |  |
| Technology characteristics (TEC) | TEC1 | Smart wearable devices provide easy-to-understand data and reports that support quick instructional decisions.  智能可穿戴设备提供易于理解的数据和报告，支持快速教学决策 | (Zhou et al., 2010; Wang et al., 2020) |
|  | TEC2 | Smart wearable devices offer stable and accurate data during physical education teaching.  智能可穿戴设备在体育教学过程中提供稳定且准确的数据 |  |
|  | TEC3 | Smart wearable devices are compatible with other teaching tools such as smartphones and tablets.  智能可穿戴设备与智能手机和平板等其他教学工具具有良好的兼容性 |  |
| Task technology fit (TTF) | TTF1 | The functions of smart wearable devices effectively meet the demands of physical education tasks.  智能可穿戴设备的功能能够有效满足体育教学任务的需求 | (Lin & Huang, 2008;Wang et al., 2023) |
|  | TTF2 | Smart wearable devices improve my efficiency in completing teaching tasks.  智能可穿戴设备提高了我完成教学任务的效率 |  |
|  | TTF3 | The functions of smart wearable devices align well with my teaching tasks, such as performance monitoring and personalized training.  智能可穿戴设备的功能与我的教学任务（如表现监测和个性化训练）高度契合 |  |
| Performance expectancy (PE) | PE1 | Using smart wearable devices helps me track students’ performance and progress more accurately.  使用智能可穿戴设备有助于我更准确地跟踪学生的表现和进步 | (Venkatesh et al., 2003;Su et al.,2024) |
|  | PE2 | Smart wearable devices significantly enhance my teaching quality and effectiveness.  智能可穿戴设备显著提升了我的教学质量和教学效果 |  |
|  | PE3 | I can assess students’ physical fitness and athletic ability more efficiently with smart wearable devices.  借助智能可穿戴设备，我能够更高效地评估学生的身体素质和运动能力 |  |
| Effort expectancy (EE) | EE1 | The interface of smart wearable devices is easy to understand and use.  智能可穿戴设备的界面易于理解和使用 | (Venkatesh et al., 2003;Su et al.,2024) |
|  | EE2 | Using smart wearable devices does not require much time or effort, and I can get started quickly.  使用智能可穿戴设备不需要太多时间或精力，我可以快速上手 |  |
|  | EE3 | I can quickly learn how to use smart wearable devices and apply them effectively in class.  我能够快速学会如何使用智能可穿戴设备，并在课堂中有效应用 |  |
| Social influence (SI) | SI1 | My colleagues encourage me to use smart wearable devices in physical education.  我的同事鼓励我在体育教学中使用智能可穿戴设备 | (Venkatesh et al., 2003;Su et al.,2024) |
|  | SI2 | My school and educational administrators support my use of smart wearable devices in teaching.  我的学校和教育管理者支持我在教学中使用智能可穿戴设备 |  |
|  | SI3 | My colleagues provide help and support for using smart wearable devices in my teaching.  我的同事在我使用智能可穿戴设备开展教学时提供帮助和支持 |  |
| Self-efficacy (SE) | SE1 | I am confident in my ability to use smart wearable devices.  我对自己使用智能可穿戴设备的能力有信心 | (Hsiao & Tang, 2024) |
|  | SE2 | I can quickly learn to use smart wearable devices even without assistance.  即使没有他人帮助，我也能快速学会使用智能可穿戴设备 |  |
|  | SE3 | I am confident in integrating the benefits of smart wearable devices into physical education.  我有信心将智能可穿戴设备的优势整合到体育教学中 |  |
| Perceived data privacy (PDP) | PDP1 | I am concerned that student information provided to smart wearable devices vendors may be misused.  我担心提供给智能可穿戴设备厂商的学生信息可能被滥用 | (Marakhimov & Joo, 2017) |
|  | PDP2 | I worry that vendors may share student information with third parties without my consent.  我担心厂商可能在未经我同意的情况下将学生信息共享给第三方 |  |
|  | PDP3 | I am concerned that student information may be used by vendors in ways I did not anticipate.  我担心学生信息可能会被厂商以我未预料的方式使用 |  |
| Continued behavioral intention to use (CBIU) | CBIU 1 | I am very interested in continuously using smart wearable devices in physical education.  我对在体育教学中持续使用智能可穿戴设备非常感兴趣 | (Wang et al., 2020; Yin et al., 2022) |
|  | CBIU 2 | I am willing to continue using smart wearable devices in my future physical education teaching.  我愿意在未来的体育教学中持续使用智能可穿戴设备 |  |
|  | CBIU 3 | I am willing to recommend smart wearable devices to my supervisors and colleagues.  我愿意向我的上级和同事推荐使用智能可穿戴设备 |  |

**References**

Zhou, T., Lu, Y., & Wang, B. (2010). Integrating TTF and UTAUT to explain mobile banking user adoption. *Computers in Human Behavior*, *26*(4), 760–767. https://doi.org/10.1016/j.chb.2010.01.013

Wang, H., Tao, D., Yu, N., & Qu, X. (2020). Understanding consumer acceptance of healthcare wearable devices: An integrated model of UTAUT and TTF. *International Journal of Medical Informatics*, *139*, 104156. https://doi.org/10.1016/j.ijmedinf.2020.104156

Lin, T.-C., & Huang, C.-C. (2008). Understanding knowledge management system usage antecedents: An integration of social cognitive theory and task technology fit. *Information & Management*, *45*(6), 410–417. https://doi.org/10.1016/j.im.2008.06.004

Wang, Z., Fang, D., Liu, X., Zhang, L., Duan, H., Wang, C., & Guo, K. (2023). Consumer Acceptance of Sports Wearables: The Role of Products Attributes. *Sage Open*, *13*(3), 21582440231182653. https://doi.org/10.1177/21582440231182653

Venkatesh, Morris, Davis, & Davis. (2003). User Acceptance of Information Technology: Toward a Unified View. *MIS Quarterly*, *27*(3), 425. https://doi.org/10.2307/30036540

Hsiao, C.-H., & Tang, K.-Y. (2024). Beyond acceptance: An empirical investigation of technological, ethical, social, and individual determinants of GenAI-supported learning in higher education. *Education and Information Technologies*. https://doi.org/10.1007/s10639-024-13263-0

Marakhimov, A., & Joo, J. (2017). Consumer adaptation and infusion of wearable devices for healthcare. *Computers in Human Behavior*, *76*, 135–148. https://doi.org/10.1016/j.chb.2017.07.016

Yin, Z., Yan, J., Fang, S., Wang, D., & Han, D. (2022). User acceptance of wearable intelligent medical devices through a modified unified theory of acceptance and use of technology. *Annals of Translational Medicine*, *10*(11), 629–629. https://doi.org/10.21037/atm-21-5510
